# Characterization of Nerve Damage After an Injury to the Adjacent Soft Tissue: A Pilot Animal Study

**DOI:** 10.1089/ten.tec.2023.0151

**Published:** 2023-12-11

**Authors:** Nesreen Zoghoul Alsmadi, Curt Deister, Nik Agrawal, Lan Tran, Rasa Zhukauskas, Debbie Neubauer Fischer, Deana Mercer

**Affiliations:** ^1^ Department of Research & Development, Axogen Corporation, Tampa, Florida, USA.; ^2^ Department of Orthopedics & Rehabilitation, University of New Mexico, Albuquerque, New Mexico, USA.

**Keywords:** nerve adhesion, nerve compression, nerve scar, peripheral nerve

## Abstract

Traumatic injuries may result in the formation of soft tissue adhesions between peripheral nerves and surrounding soft tissue. These soft tissue adhesions lead to compression and ischemic stress within fascicles due to nonpliability of adhered scar tissue, and nerve tension due to loss of nerve gliding from scar tethering. These changes in the soft tissue bed surrounding the nerve may result in axon degeneration and neuroma-in-continuity. Preclinical models that simulate clinically relevant levels of scar in the nerve environment may be impactful to the development of surgical techniques and treatments to prevent adhesions. This study presents the results of a rodent model with an induced indirect nerve injury by (1) thermal insult to the soft tissue bed surrounding the nerve and (2) air-drying the surrounding soft tissue bed of the nerve. Our findings suggest that inducing an injury of the soft tissue bed results in increased intraneural scar and extraneural adhesions to the nerve compared to a sham procedure. Thermal induced injuries showed more macrophages and changes in nerve health compared to air-dried induced injuries. The changes in the nerves of the induced injury groups, specifically the thermal injury group, may be meaningful for evaluating treatments for nontransected nerve injuries.

**Impact statement:**

This study provides both quantitative and qualitative assessments of adhesions, scar, and associated nerve damage in a rat model. Development of these animal models may provide methods for assessing treatments aimed at preventing adhesions and scar tissue formation in peripheral nerves.

## Introduction

Traumatic injuries or nerve manipulation during surgery may result in soft tissue adhesions and scar formation between the nerve and surrounding tissue, which may lead to loss of nerve function (sensation and/or motor function), neuropathic pain, and altered quality of life.^1,2^ Extraneural scarring and adhesions may be associated with ischemia, intraneural scar, and nerve contracture.^2–6^ It can also lead to tension on the nerve from tethering, causing neuropathic pain.^2–6^ The compression, tension, and ischemic stress within individual fascicles associated with extraneural adhesions may affect axon mobility, which may lead to axon degeneration, disorganized regeneration, and formation of a neuroma-in-continuity.^6,7^

This tissue injury and scarring process has been evaluated by either creating a direct lesion on the intact nerve or an indirect lesion to the surrounding soft tissue bed.^8^ Described animal models of intact peripheral nerve damage include nerve ligature, nerve freezing with cryoneurolysis, injection-induced inflammatory neuritis, and thermal/laser nerve injury.^9^ For example, exposing a rat sciatic nerve and surrounding soft tissues to a glue containing glutaraldehyde has been used to evaluate nerve adhesions, macrophages, Schwann cells, axons, and nerve function.^10^ The results demonstrated inflammation and scar within the nerve, axon degeneration, scar formation around the nerve, and functional deficits.^10^ Indirect lesion models using thermal injury or abrasion to the soft tissue bed surrounding the nerve have been shown to result in extraneural scarring.^11^

While various nontransected nerve models have been utilized, most animal models utilize difficult to replicate techniques, lack control of the induced injury effects, or cause unintentional comorbidities (e.g., autonomy).^12^ In this study, we pursued development of an easy to replicate model of indirect nerve injury using a thermal technique^13^ and air-drying technique,^14^ as these previously described techniques seemed to be the easiest to implement and had the potential to be the most reproducible. We hypothesized that an induced injury to the soft tissue surrounding the nerve using thermal injury or air-drying would produce measurable nerve injury and changes to clinically relevant nerve morphology. These findings provide evidence of the cellular and structural changes that occur within and around the nerve after these two types of injury to the surrounding soft tissues.

## Methods

Eighteen male Lewis rats 8–9 weeks of age and weighing 200–300 g were used in this study. All animal experiments were approved by the University of South Florida (USF) Institutional Animal Care and Use Committee and were performed in accordance with the standard of care practices at the USF Morsani College of Medicine and Heart Institute. Animals were randomly assigned to a group, as follows: (1) Sham group (*n* = 6), (2) air-dried injury group (*n* = 6), or (3) thermal injury group (*n* = 6).

### Surgical procedures

All surgical procedures were performed under aseptic conditions, and all animals were prepared for surgery in a dedicated area separate from the surgical field. Rats were exposed to isoflurane, and the pedal withdrawal reflex was used to determine the depth of anesthesia. For all groups, the sciatic nerve was exposed and circumferentially released from the surrounding soft tissues at the mid-thigh level using surgical neurolysis. The sham group was immediately closed after exposure of the sciatic nerve using standard surgical wound closure techniques.

The air-dried and thermal injuries were prepared similarly. Immediately after, the sciatic nerve was exposed and circumferentially released from the surrounding tissue at mid-thigh level. An area measuring ∼10 × 20 mm was demarcated for the predetermined injury, called the injury site, ∼7 mm from the iliac fascia. The nerve was wrapped with a saline-moistened alginate foam (#A6209; Simpurity Foam, Safe N Simple) and an esmark bandage (#388763; Mckesson), which protected the nerve during the injury and was used for gentle nerve manipulation and retraction during the tissue bed preparation. The soft tissue surrounding the injury site was covered with saline-moistened alginate foam and an esmark bandage to protect the area from damage. Care was taken to prevent over manipulation or traction on the nerve. The soft tissue of each injury site was then exposed to either an air-dried injury or a thermal injury.

The air-dried injury was created with forced air using an air pump (Model #AP58; Vivosun), which was set at 45% of maximum air flow. The air pump tip was placed at a 90° angle above the injury site and continually moved to evenly expose the soft tissue bed surrounding the nerve to forced air for a period of 20 min. The thermal injury was created using a bipolar coagulator (Model #60-5600-002; CONMED), with the power set to 35 watts. The tips of the forceps were held ∼3 mm apart at a 45° angle on the edge of the injury area. The bipolar coagulator was powered for 3 s. The forceps were moved to an adjacent area within the injury region. This process was repeated until the entire premeasured and marked injury area was exposed to thermal injury.

Once the injury was completed, the nerve was placed in its original position in the central region of the injury area. The protective coverings were removed, and the area was irrigated with sterile saline. The surgical site was closed using standard surgical wound closure techniques. Daily postoperative monitoring included inspection for signs of pain and infection until the study endpoint.

### Endpoint procedures

Rats were euthanized 6 weeks post surgery. A 6-week time point was selected to allow deposition of collagenous tissues, which typically occurs between 3 and 8 weeks depending on the vascularity and characteristics of the injured tissue.^15^ Endpoint procedures were completed, including bodyweight measurements, gross evaluation of surgical site adhesions, and wet gastrocnemius muscle weight (both the induced injury leg and contralateral uninjured leg).

The surgical site adhesion assessment was conducted during the nerve explant with minimal neurolysis applied to the top of the nerve and no neurolysis performed on the side or the back of the nerve. The degree of adhesions surrounding the nerve was assessed for the sham and induced injury surgical sites using a semiquantitative scale. This semiquantitative scale included assessment of nerve immobility, adhesion quality, tenacity, and extent of site involvement for adhesions (Table 1). Adhesions were evaluated using scoring similar to criteria previously described by Kokkalis et al.^16^ The individual adhesion criteria scores were summed for the total adhesion score (ranging from 1 to 13). A single blinded evaluator performed all assessments.

**Table 1.** Semiquantitative Scale for Scoring Soft-Tissue Adhesion Surrounding the Nerve

Assessment	Description		Point value
Nerve immobility (max = 2)	No immobility: Similar to a contralateral uninjured nerve		0
	Movement is restricted by scar tissue		1
	Completely immobilized by scar tissue		2
Adhesion quality^a^ (Circle all that apply, do not add the scores, highest score is selected and recorded, max = 4)	None: Similar to a contralateral uninjured nerve		0
	Appearance	Transparent	1
		Translucent	2
		Opaque	3
	Vascularization	Avascular	1
		Capillaries present	3
		Large blood vessels present	4
Tenacity^a^ (max = 3)	None: Similar to contralateral uninjured nerve		0
	Adhesions fall apart with blunt dissection		1
	Adhesions tear with significant traction		2
	Adhesions require sharp transection		3
Extent of site involvement for adhesions^b^ (max = 4)	None: Similar to contralateral uninjured nerve		0
	<50%		2
	>50%		4
Total adhesion score	Add the highest score from each assessment criteria above (nerve immobility, adhesion quality, tenacity, and extent of site involvement for adhesions)		1–13

^a^ Scores adapted from Kokkalis et al.^16^ include adhesion quality, tenacity, and extent of site involvement for adhesions. Scores for adhesion quality were split into two categories, appearance and vascularization. Vascularization scores did not include a point value of “2” because avascular corresponded to a score of “1” and “2” in Kokkalis et al.^16^; therefore, only a score of “1” was recorded for avascular.

^b^ Scores for extent of site involvement for adhesions were categorized in 50% increments. Previously outlined scoring by Kokkalis et al.^16^ were categorized in 25% increments. The evaluation of adhesion extent in 25% increments was difficult to differentiate; therefore, we used 50% increments and removed the scores of “1” and “3.”

The gastrocnemius muscles were collected and weighed from both the surgical leg and contralateral uninjured leg of each rat. Wet gastrocnemius muscle weight was recorded as a percent weight (Muscle weight % = wet weight of the gastrocnemius muscle of the leg with an induced injury/wet weight of the gastrocnemius muscle of the contralateral uninjured leg). To collect histology samples of the nerve, the sciatic nerve was exposed using standard surgical procedure.

### Histology

Both the nerve and the surrounding muscle were collected during histology sample explant. The tissues included 2 mm proximal to the injury site, the injury site, and 5 mm distal to the injury site. In addition, 17 mm of nerve from the contralateral leg was recovered from six animals to serve as an uninjured nerve control. The specimens were marked with a tissue marker at the proximal stump and immersed in 10% neutral-buffered formalin for 48 h. Tissues were embedded in paraffin and transversely cut into 5-μm-thick sections. Paraffin sections were deparaffinized in xylene and rehydrated in graded alcohol solutions. The following staining was performed: Hematoxylin and eosin (H&E), Masson's Trichrome (MT), Picrosirius Red (PSR), and Luxol Fast Blue (LFB).

Immunohistochemistry slides were also prepared in the following manner after sectioning and deparaffinizing: Heat-mediated antigen-retrieval was performed by placing the slides in sodium citrate buffer (pH6; Abcam). Peroxidase activity and nonspecific protein binding were quenched using Bloxall (Abcam) and 10% normal goat serum with 1% bovine serum albumin in tris buffered saline, respectively. Slides were then stained with antibodies to one or more of the following: CD68 (ab125212, 1:500; Abcam), Myelin Basic Protein (MBP, ab40390, 1:100; Abcam), or Neurofilament (NF, N2787, 1:200; Sigma). For CD68 staining, incubation with goat anti-rabbit immunoglobulin G heavy and light chains (Horseradish Peroxidase) secondary antibody was followed by exposure to diaminobenzidine (DAB) (BDB550880; Fisher Scientific) to visualize immunoreactivity. For NF and MBP, a fluorescent secondary antibody (Fisher Scientific, #PIA32731 for Alexa Fluor™ Plus 488 and #PIA3274 for Alexa Fluor™ Plus 594) was used at 1:400 dilution. All DAB immunohistochemistry sections were counterstained in hematoxylin, dehydrated, and cover slipped.

H&E-, MT-, LFB-, CD68-, MBP-, and NF-stained slides were scanned using a Zeiss Axio Scan Z1 automated slide scanner at 400 × magnification using the bright-field light source. NF and MBP double-stained slides were scanned using a Zeiss Axio Scan Z1 automated slide scanner at 400 × magnification using the fluorescent light source. The Zeiss Axio Scan Z1 automated slide scanner rendered a single image for each histology section. Slides stained with PSR were imaged using an Olympus BX41 microscope, using the bright-field light source with a polarizing filter applied over the light source. Images were collected for the entire sample cross-section.

Histologic quantification was performed using ImageJ (FIJI, National Institutes of Health) for collagen:cell ratio, percent macrophage area, extraneural collagen area, and the number of myelinated axons within the nerve. The overall morphology and presence of cells were evaluated manually using H&E slides. Collagen deposition and organization were assessed using MT and PSR slides. Image analysis of MT slides was performed to evaluate collagen to cell ratio (collagen:cell ratio) by adjusting the hue, saturation, and brightness levels of each image to capture the relevant blue area that corresponded to the collagen.

Using the same function, the manual color threshold was adjusted to select the pink areas that corresponded to cellular cytoplasm. An average collagen to cell ratio was obtained by dividing the collagen pixel area by the cellular cytoplasm pixel area for each image. Image analysis of PSR slides was performed to evaluate the extraneural collagen deposition surrounding the nerve, which was measured by color thresholding polarized light PSR images for red, yellow, and green colors. Histology samples with distorted architecture were removed from quantitative analysis. Sample numbers were recorded for each analysis and presented in graphs.

Myelinated axons were counted in three randomly selected 100 × 100 μm areas of the LFB-stained slide images. The reported value for each sample was an average of the counts from the three areas evaluated. Macrophages were quantified using CD68 stained slides, and identified by color thresholding for brown areas corresponding to anti-CD68-stained areas. This area was normalized by the total nerve area for each image. The combined MBP- and NF-stained slides were qualitatively assessed for peripheral nerve health.

### Statistical analysis

Study populations were assessed for normal distribution before statistical evaluation. Each dependent measure was analyzed using one-way analysis of variance (ANOVA) and Tukey post-test analyses were used when main effect differences were statistically significant. Groups were considered significantly different when *p* ≤ 0.05, adjusted for multiple comparisons, as appropriate. Results are reported as mean ± standard deviation and 95% confidence interval (95% CI).

## Results

**FIG. 1.**
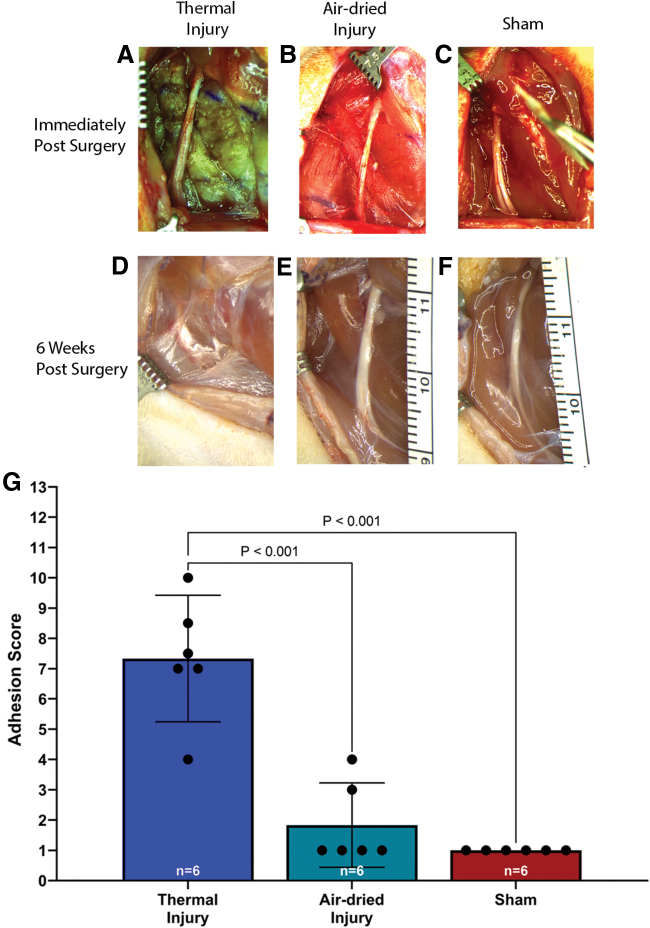
Representative images of the sciatic nerve immediately after surgery and before closing the surgical site in **(A)** thermal injury, **(B)** air-dried injury, and **(C)** sham groups. Representative images of the sciatic nerve 6 weeks after surgery in the **(D)** thermal injury, **(E)** air-dried injury, and **(F)** sham groups. **(G)** Adhesion scores were significantly higher in the thermal tissue injury group compared to the air-dried injury and sham groups, *p* < 0.001. There was no significant difference between the air-dried injury and sham groups. Data presented as mean and 95% confidence interval (95% CI). Color images are available online.

Six weeks after injury, the nerves were covered with fibrous connective tissue that varied in severity (Fig. 1A–F). A one-way ANOVA revealed a significant effect of injury on the adhesion score, *F*(2, 15) = 5.703, *p* = 0.01. Tukey's post-test indicated that the mean value of the degree of adhesions was significantly higher in the thermal tissue injury group (mean 7.3 ± 2.0, 95% CI: 5.2–9.4; Fig. 1G) compared with those in the air-dried tissue injury group (mean 1.8 ± 1.3, 95% CI: 0.4–3.2) and the sham group (mean 1.0 ± 0.0, 95% CI: 1.0–1.0), *p* < 0.001.

**FIG. 2.**
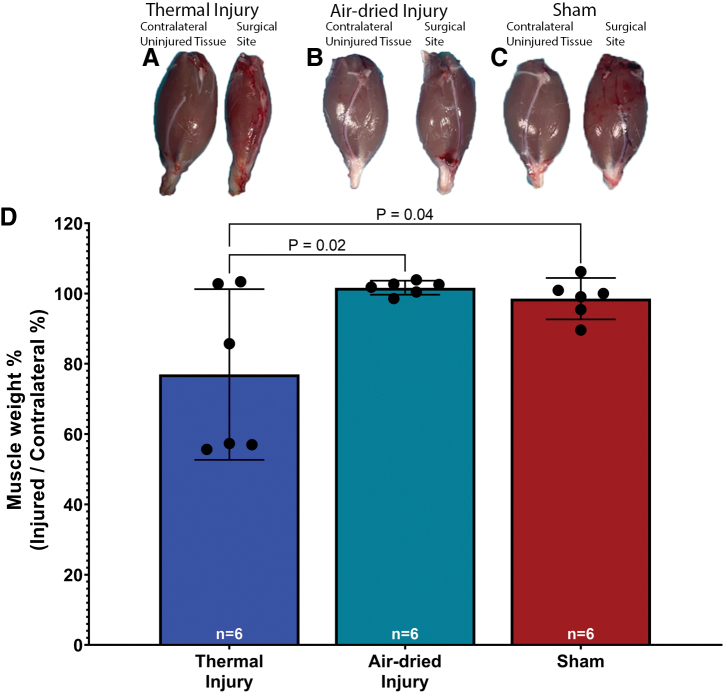
**(A)** There was visible muscle atrophy in the gastrocnemius muscle explants of the thermal injury group. The **(B)** air-dried injury and **(C)** sham groups showed no visible muscle atrophy. **(D)** The percent muscle weight was significantly lower in the thermal tissue injury group than the sham and air-dried groups, *p* = 0.02 and *p* = 0.04, respectively. These findings indicate muscle atrophy in the thermal injury group. Data presented as mean and 95% CI. Color images are available online.

A one-way ANOVA revealed a significant effect of injury on the wet weight of the gastrocnemius muscle, *F*(2, 15) = 37.18, *p* < 0.001. Tukey's post-test indicated that the mean value of the wet weight of the injured gastrocnemius muscle, expressed as a percent of the contralateral uninjured gastrocnemius muscle, was significantly lower in the thermal injury group (mean 77.0% ± 23.1%, 95% CI: 52.7–101.3, Fig. 2A, D) than in the air-dried tissue injury (mean 101.7% ± 1.9%, 95% CI: 99.7–103.7, Fig. 2B) and sham groups (mean 98.5% ± 5.6%, 95% CI: 92.7–104.4, Fig. 2C), *p* = 0.02 and *p* = 0.04, respectively. These significant differences indicate muscle atrophy in the thermal injury tissue group.

### Histological evaluation of nerve

**FIG. 3.**
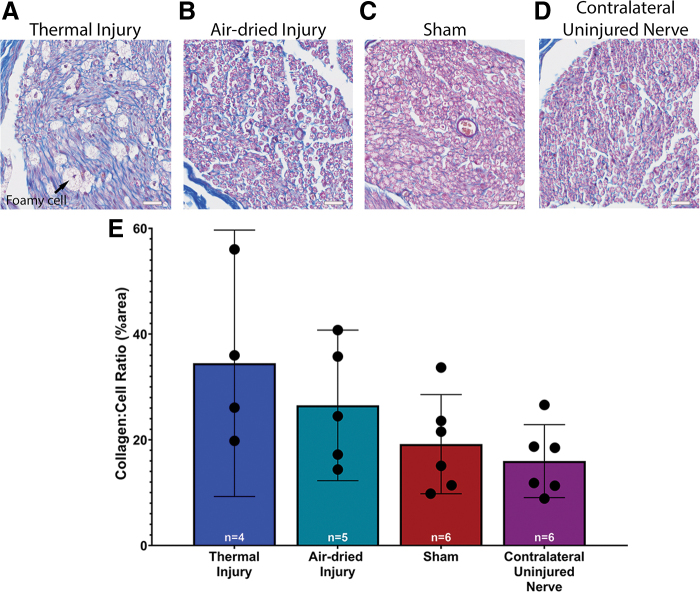
Representative images of the cross-section of the sciatic nerve with Masson trichrome 6 weeks postsurgery: **(A)** Thermal injury. Foamy phagocytes were identified in the thermal tissue injury group, indicated by the *black arrow*. **(B)** Air-dried injury, **(C)** sham, and **(D)** contralateral uninjured nerve. Scale is 20 μm for all images. **(E)** Quantitative assessment of intraneural collagen showed that the intraneural scar trended highest in the thermal injury group, and lowest in the contralateral uninjured nerve. Data presented as mean and 95% CI. Color images are available online.

A one-way ANOVA revealed no significant effect of injury on the collagen to cell ratio, *F*(3, 17) = 2.905, *p* = 0.06. While not statistically significant, there was a trend showing that the collagen to cell ratio was highest, on average, in the thermal injury group (34.5% ± 15.8%, 95% CI: 9.3–59.68, Fig. 3A–E) and lowest in the contralateral uninjured nerve group (16.0% ± 6.6%, 95% CI: 9.1–22.9; Fig. 3D).

Foamy phagocytes were also noted in the thermal injury group. These foamy phagocytes were identified by their foamy appearance, a result of macrophages that have engulfed lipids.^17^ These foamy phagocytes are considered markers of Wallerian degeneration.^17–19^

**FIG. 4.**
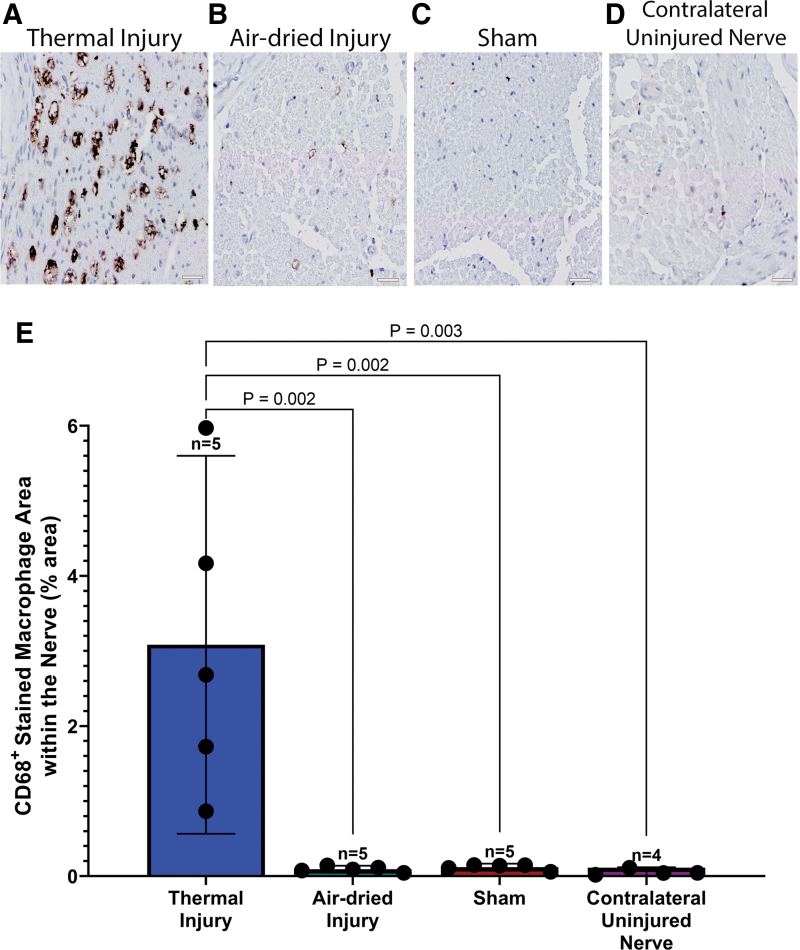
Representative images of the sciatic nerve 6 weeks postsurgery, stained with CD68 in **(A)** thermal injury, **(B)** air-dried tissue injury, **(C)** sham, and **(D)** contralateral uninjured nerve groups. There were more CD68^+^ cells in the thermal injury group compared to all other groups. Scale is 20 μm for all images. **(E)** Quantitative assessment showed that the thermal injury group showed significantly higher presence of CD68^+^-stained macrophages compared to all other groups, *p* = 0.002 for the air-dried and sham groups and *p* = 0.003 for the contralateral uninjured nerve group. Data presented as mean and 95% CI. Color images are available online.

The thermal injury group showed a notable presence of macrophages positive for CD68 (Fig. 4A), which was not apparent in the remaining groups (Fig. 4B–D). A one-way ANOVA revealed a significant effect of injury on the population of CD68^+^-stained macrophages, *F*(3, 15) = 10.01, *p* < 0.001. Tukey's post-test indicated that the mean value of CD68-positive macrophages showed a significantly higher percent area in the thermal tissue injury group (3.1% ± 2.0%, 95% CI: 0.6–5.6, Fig. 4E) compared to all other groups, *p* = 0.002 for the air-dried (0.09% ± 0.04%, 95% CI: 0.05–0.14) and sham (0.12% ± 0.04%, 95% CI: 0.07–0.17) groups and *p* = 0.003 for the contralateral uninjured nerve group (0.05% ± 0.04%, 95% CI: 0.009–0.12).

**FIG. 5.**
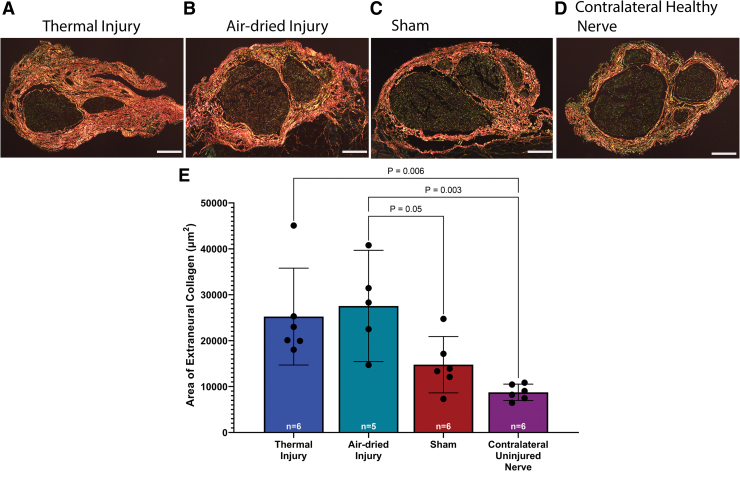
Representative images of PSR-stained samples of sciatic nerve 6 weeks postsurgery in **(A)** thermal injury, **(B)** air-dried injury, **(C)** sham, and **(D)** contralateral uninjured nerve groups. **(E)** Quantitative assessment of the area of collagen birefringence showed significantly more extraneural collagen in the air-dried injury compared to the sham and contralateral uninjured nerve (*p* = 0.05 and *p* = 0.003, respectively). In addition, there was significantly more extraneural collagen in the thermal injury group compared to the contralateral uninjured nerve group (*p* = 0.006). Data presented as mean and 95% CI. Scale bar is 200 μm in all images. PSR, picrosirius red. Color images are available online.

Extraneural collagen deposition, or extraneural scar, was most abundant surrounding the nerve of the thermal and air-dried injury groups (Fig. 5A–D). A one-way ANOVA revealed a significant effect of injury on extraneural scarring, *F*(3, 19) = 7.90, *p* = 0.001. Tukey's post-test indicated that the mean area of extraneural collagen was significantly greater in the thermal injury group (25,240 ± 10,051 μm^2^, 95% CI: 14,692–35,787 μm^2^) compared to the contralateral uninjured nerve group (8742 ± 1700 μm^2^, 95% CI: 6958–10,526 μm^2^, *p* = 0.006, Fig. 5E).

In addition, there was significantly more extraneural collagen in the air-dried injury group (27,547 ± 9760 μm^2^, 95% CI: 15,429–39,666 μm^2^) compared to the sham (14,759 ± 5843 μm^2^, 95% CI: 8627–20,891 μm^2^) and contralateral uninjured nerve, *p* = 0.05 and *p* = 0.003, respectively. There was no significant difference between the thermal injury group and the sham group, *p* = 0.11, which may be attributable to a lower mean difference between the thermal injury and sham groups.

**FIG. 6.**
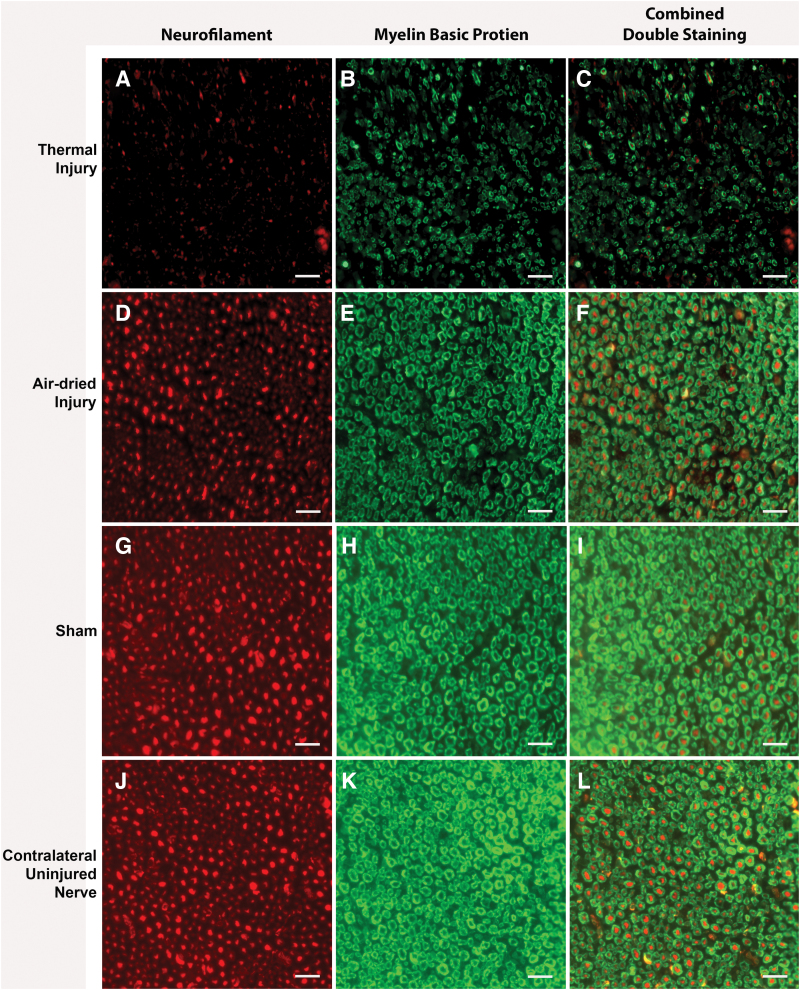
Representative immunohistochemical double-staining images of NF (*red*) and MBP (*green*) for **(A–C)** thermal injury, **(D–F)** air-dried injury, **(G–I)** sham, and **(J–L)** contralateral uninjured nerve group. Qualitatively, there were smaller myelin sheaths and axons in the thermal tissue injury group, which is evidence of nerve damage. Scale bar is 20 μm for all images. MBP, Myelin Basic Protein; NF, neurofilament. Color images are available online.

Qualitatively, the double-stained NF and MBP fluorescent immunohistochemistry images showed that the thermal tissue injury group exhibited myelin demarcated axons of variable sizes, indicating nerve injury (Fig. 6A–C). The myelin and NF staining in the thermal injury appeared less robust compared to the air-dried injury, sham, and contralateral uninjured nerve (Fig. 6D–L).

**FIG. 7.**
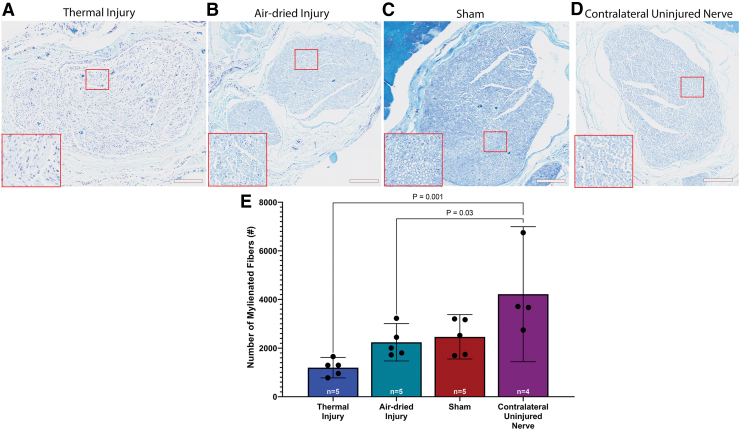
LFB staining 6 weeks postsurgery **(A–D)** shows myelin disruption and myelin loss in the **(A)** thermal injury group. Myelin disruption was not visibly apparent in the **(B)** air-dried injury, **(C)** sham, and **(D)** contralateral uninjured nerve groups, suggesting healthy myelin sheaths in these groups. Scale bars for all images are 200 μm. **(E)** The number of myelinated fibers was significantly lower in the thermal tissue injury and air-dried injury groups compared to the contralateral uninjured nerve, *p* = 0.001 and *p* = 0.03, respectively. This indicated myelin sheath damage in the thermal and air-dried injury groups. Data presented as mean and 95% CI. LFB, Luxol Fast Blue. Color images are available online.

A one-way ANOVA revealed a significant effect of injury on the number of myelinated fibers, *F*(3, 15) = 7.75, *p* = 0.002. Tukey's post-test indicated that the mean value of myelinated fibers in the contralateral uninjured nerve (4217 ± 1746 fibers, 95% CI: 1438–6995 fibers, Fig. 7D, E) was significantly higher than the thermal injury group (1194 ± 340, 95% CI: 773–1616 fibers, Fig. 7A, E) and the air-dried injury group (2238 ± 618 fibers, 95% CI: 1471–3006 fibers, Fig. 7B, E), *p* = 0.001 and *p* = 0.03, respectively. There was no significant difference between the sham (2463 ± 734 fibers, 95% CI: 1553–3374 fibers, Fig. 7C, E) and any other group.

## Discussion

Peripheral nerves are capable of regeneration after injury; however, extraneural and intraneural scar formation can hinder the regeneration process.^20,21^ Scarring has been noted as an inevitable result of the peripheral nerve degeneration and regeneration process.^21^ Intraneural scarring may lead to shrinkage of endoneurial tube sheath diameter by up to 80–90% at 3 months after an injury.^21,22^ This scarring is progressive from the extraneural tissues to the intraneural tissues.^7^ Extraneural adhesions lead to scar over the epineurium, which ultimately results in scar tissue formation within the connective tissue of the nerve.^7^

Scarring of the connective tissues surrounding vasculature and fascicles leads to reduction in neural blood flow, which causes intrafascicular swelling, myelin sheath thinning, and Wallerian degeneration.^7^ This nerve degeneration or scar tissue formation within and around an intact nerve may be caused by trauma, surgery, repetitive motion, or systemic diseases (e.g., diabetes mellitus). Animal models used to evaluate nerve degeneration and scar tissue formation in peripheral nerves after an injury often lack reproducibility with clinically meaningful outcome measures.^23^ We hypothesized that an induced injury to the soft tissue surrounding the nerve using thermal injury or air-drying would result in histologic changes to clinically relevant nerve morphology.

Our study showed that the induced injury groups showed more extraneural and intraneural collagen deposition and macrophage population compared to the sham and contralateral uninjured nerve. Gross evaluation showed that there was a significantly higher degree of extraneural adhesions in the soft tissue bed surrounding the sciatic nerve in the thermal injury group compared to the air-dried injury and sham groups. Histological examination of PSR-stained slides showed that there were significantly higher areas of extraneural collagen, or extraneural scarring, in the thermal injury and air-dried injury groups compared to the contralateral uninjured nerve. Extraneural scarring was also significantly higher in the air-dried injury group compared to the sham group. These results indicate that the neurolysis alone in the sham procedure caused less extraneural scarring compared to neurolysis with an induced injury in the air-dried and thermal injury groups.

Intraneural collagen deposition, or intraneural scarring, trended highest in the thermal injury group, followed by the air-dried injury group, sham, and lowest in the contralateral uninjured nerve group. There was no significant difference between groups; however, this trend indicated that the induced injury groups exhibited more signs of intraneural scarring than the sham and contralateral uninjured nerve groups. It should be noted that the intraneural scarring in the induced injury groups was highly variable across samples and future studies should account for this in their experimental design.

Further signs of intraneural tissue disruption were evident due to the presence of foamy phagocytes noted in the thermal injury group. In addition, quantitative assessment of the presence of CD68^+^ macrophages showed significantly more macrophages in the thermal injury group compared to the air-dried injury group, sham and contralateral uninjured nerve. The percent area of CD68-positive macrophages and the presence of foamy phagocytes in the thermal injury group indicate that there was a substantial injury, which initiated Wallerian degeneration. There were little to no CD68-positive macrophages or foamy cells in the air-dried injury and sham groups, which suggests there was no substantial nerve damage in these groups.

Qualitative assessment of immunohistochemical double-staining of NF and MBP showed a less robust signal in the thermal injury group compared to all other groups, indicating indirect nerve damage because of thermal injury to the surrounding muscle bed. Quantitative assessment found fewer myelinated fibers in the air-dried and thermal injury groups compared to the contralateral uninjured nerve.

However, there was no significant difference between the induced injury groups and the sham group. The nonsignificant difference between the induced injury groups and the sham group may indicate that even simple exposure and neurolysis of the nerve can result in some extent of nerve injury; however, additional studies are needed to determine if these similarities exist with repeated studies. Gross weight of the gastrocnemius muscle further confirmed a higher degree of nerve damage in the thermal injury group, as the gastrocnemius muscle weight of the thermal injury group was significantly lower than the air-dried injury or sham groups.

This study has shown that nerves adhere to the surrounding tissue bed in varying degrees depending on the severity of insult to the surrounding nerve tissue bed. The sham group, which simulates manipulation of a nerve during surgical exposure, was not free of adhesions. The thermal group had extensive scarring, which restricts nerve gliding capability. In preclinical models, wrapping a nerve has been shown to reduce postneurolysis peripheral nerves from developing adhesion and function loss by effectively blocking scarring and preventing adhesion-related damage in the peripheral nerves.^24^

Clinically, attempts to protect the nerve from the surrounding environment have included autologous wraps, such as vein, and implantable off-the-shelf biomaterials.^25^ While each of these wrapping materials has their advantages and disadvantages, clinical findings suggest that wrapping a nerve after neurolysis results in improvements in objective and subjective clinical outcome measures.^25^ In future studies, the thermal injury model described in this study may be used to evaluate the effects of various nerve wrapping techniques and materials. These materials could potentially be used to prevent nerve adhesions and is a future direction of this work.

Limitations of this study include a lack of functional testing (e.g., walking track analysis, electrophysiology) and quantification of foamy phagocytes. While functional testing is beneficial for the evaluation of peripheral nerve damage, the focus of this study was the development of histological assessments of clinically relevant nerve morphology. Future studies should consider including functional testing, as this study indicated that nerve damage was present in the thermal injury group.

Foamy phagocytes are indicative of Wallerian degeneration,^19^ which was not an expected response due to the injury being limited to the soft tissue surrounding the nerve. While the presence of suspected foamy phagocytes was noted in the histology of thermal injury sections, these cells were not specifically evaluated in this study. Future studies should quantitatively evaluate Wallerian degeneration in this model of induced indirect nerve injury. Despite these limitations, this study describes a reproducible method to create scar tissue formation in and around the peripheral nerve without multiphased surgeries. Future studies should be conducted to evaluate the impact of different wrapping materials or techniques in this induced thermal injury scar model.

## Conclusion

This pilot animal study described two methods of induced indirect nerve injury. While the n-value for each study arm is low, there were statistical differences indicating that induced indirect nerve injuries could create measurable differences in nerve scarring, adhesion, and nerve health measures.

Our findings suggest that injury to the soft tissue bed surrounding a nerve can result in both extraneural and intraneural scarring and nerve damage. In addition, an indirect thermal injury was more severe and resulted in a higher degree of inflammation and myelin disruption, and a significant loss of gastrocnemius muscle mass. In addition, the presence of foamy phagocytes in the thermal injury group indicated that the nerve may undergo Wallerian degeneration after the application of heat to the soft tissue surrounding a nerve. Therefore, the indirect thermal injury model may be used in future studies aimed at examining the effects of nerve protection treatments.
